# The impact of cutaneous neurofibromas on quality of life and mental health in neurofibromatosis type 1

**DOI:** 10.1111/1346-8138.17276

**Published:** 2024-06-24

**Authors:** Brieana Dance, Alice Dardare, Jane Fleming, Sue‐Faye Siow, Timothy E. Schlub, Hilda Crawford, Rebecca B. Saunderson, Claire Wong, Yemima Berman

**Affiliations:** ^1^ Department of Clinical Genetics Northern Sydney Local Health District Sydney New South Wales Australia; ^2^ Northern Clinical School, Faculty of Health and Medicine University of Sydney Sydney New South Wales Australia; ^3^ Sydney School of Public Health, Faculty of Medicine and Health University of Sydney Sydney New South Wales Australia; ^4^ Department of Dermatology Northern Sydney Local Health District Sydney New South Wales Australia

**Keywords:** mental health, neurofibromatosis, patient reported outcome measures, quality of life, treatment

## Abstract

The skin manifestations of neurofibromatosis 1 significantly reduce health‐related quality‐of‐life. However, data on the utility of existing surveys in capturing neurofibromatosis 1 skin treatment outcomes are lacking. This quantitative study examined the relationship between clinician‐rated severity and visibility and patient‐rated itch and quality‐of‐life (QoL) to (1) establish baseline levels of skin‐ and condition‐specific‐related QoL, itch, depression and anxiety; (2) identify patient concerns to inform the development and evaluation of skin interventions; and (3) compare the sensitivity of different QoL measures. Validated scales included Skindex‐29, Dermatology Life Quality Index (DLQI), Neurofibromatosis 1‐adult quality‐of‐life (NF1‐AdQOL) questionnaire, and the Hospital Anxiety and Depression Scale (HADS). We recruited 100 participants (response rate: 95%). Of these, 42% reported itch and 23% had probable clinical anxiety. Our cohort had higher levels of anxiety and total HADS scores compared to a control population. Using multivariate regression analysis, increasing visibility significantly predicted poorer QoL using the Skindex‐29, NF1‐AdQOL, and DLQI (*p* < 0.05); and itch significantly predicted worse QoL in Skindex‐29 and NF1‐AdQOL (*p* < 0.05). The highest mean scoring questions in Skindex‐29 and NF1‐AdQOL concerned worry about worsening skin disease and embarrassment. The highest mean scoring questions in DLQI were regarding itch, pain, and embarrassment. Items asking specifically about cutaneous neurofibromas (cNF) scored higher than comparable skin‐specific questions (*t*‐test *p* value <0.05). In summary, this study provides insights into the factors contributing to impaired QoL, anxiety, and mood in NF1 patients with cutaneous neurofibromas. Key factors identified for use in cNF measures include visibility, itch, anxiety, embarrassment, fears of worsening skin disease, and cNF‐specific questions.

## 1 INTRODUCTION

Neurofibromatosis type 1 (NF1 OMIM:162200), is a progressive, autosomal dominant tumor predisposition disorder.^1^ NF1 is associated with a significant disease burden and reduced quality‐of‐life (QoL).^2^, ^3^, ^4^, ^5^, ^6^, ^7^, ^8^, ^9^ Prognosticating skin disease severity outside of two variants, *NF1*:p.Met992del and a missense change at *NF1*:p.Arg1809, which lack cutaneous neurofibromas (cNF), and the recurrent 1.4 Mb NF1 microdeletion (Type 1), which is associated with a more severe (cNF) burden, is challenging.^10^, ^11^

Despite the potential for serious systemic complications, such as malignancy, the principal concern for younger adults with NF1 is the burden of cutaneous manifestations.^3^ NF1 patients frequently present for medical care seeking treatment of their cNFs.^12^ Previous studies have found higher rates of depression: up to 55%, in NF1 individuals compared to the general population,^6^, ^13^ and 15% of NF1 patients had clinically significant anxiety.^6^ NF1 disease visibility has been associated with psychiatric morbidity,^2^, ^6^, ^14^, ^15^, ^16^, ^17^, ^18^, ^19^ although this has not been studied in the context of patients seeking treatment for their cNFs.

Itch is increasingly being recognized as another troublesome health concern in NF1, affecting up to 35% of patients daily^20^, ^21^ and up to 70% of patients “almost daily” or “most days”.^16^, ^21^ NF1‐related itch has recently been found to predict reduced QoL.^16^ The impact of NF1‐related itch on levels of anxiety and depression has not been studied. Subsequently, there is a risk of under‐recognition and under‐treatment of itch by clinicians.

With the use of laser, electrodessication, surgical excision, and medical interventions to reduce cNF burden and itch,^22^, ^23^, ^24^ there is a need to establish internationally standardized measures of treatment success.^25^, ^26^ However, given the inherently personal nature of how an individual responds to aesthetic changes, patient reported outcome measures (PROMs) are equally important. Multiple QoL tools exist including tools that are dermatology‐ and NF1‐specific.^27^, ^28^, ^29^, ^30^, ^31^ Indeed, a few NF1‐specific tools (the impact of NF1 on quality‐of‐life questionnaire [INF1‐QoL] and the Pediatric Quality of Life Inventory [PedsQL] NF1 adult module) have been identified for possible use in the adult population; however, limitations include restricted use and psychometric testing for the INF1‐QoL and large item numbers for PedsQL‐NF1.^32^ A cNF‐specific QoL has been validated in a French and American patient cohort, but it has not been studied longitudinally or used to evaluate cNF treatments.^33^ Therefore, it is important to identify the best skin‐, NF1‐ or cNF‐related Qol items to be used to assess cNF treatment outcomes.

The primary aim of this study was to establish baseline levels of skin‐ and NF1‐related QoL, itch, depression, and anxiety in patients with NF1 presenting for treatment for cNFs at an NF1 skin clinic using a variety of commonly used QoL measures. A secondary aim was to identify important patient concerns to inform the development and evaluation of skin interventions for individuals with NF1. Third, we sought to compare the sensitivity of different validated scales to patient concerns and clinical factors, to inform the utility of existing survey tools in NF1.

## 2 METHODS

A cross‐sectional cohort study examined the health and QoL of adults with NF1 attending a clinical genetics and dermatology clinic in Australia. Laser treatments and surgical excisions of primarily cNFs are performed at this clinic. This research was approved by the Northern Sydney Local Health District Ethics Committee (reference 2019/ETH08177). All participants provided written informed consent.

### 2.1 Participants and recruitment

NF1 patients attending the NF1 skin clinic (July 2015–August 2021) were invited to participate in the study if they were >18 years of age and able to provide informed consent and complete surveys written in English. For adult patients with an intellectual disability a parent/guardian completed a survey on their behalf.

### 2.2 Instrumentation

We used the skin‐specific QoL survey, Skindex‐29 (30 items) (Mapi Research Trust, Lyon, France), and the NF1‐specific QoL survey, NF1‐AdQOL (31 items), that measure the impact of disease on QoL within three domains: physical symptoms, emotions and functioning.^28^, ^30^ [*Skindex‐29 contact information and permission to use: Mapi Research Trust, Lyon, France*]. Responses were converted to a linear scale from 0 (no effect) to 100 (effect all the time). The 10‐item Dermatology Life Quality Index (DLQI), which assesses five domains (feelings, daily activities, leisure, work/school, personal relationships, and treatment) was added in May 2016.^29^ Each question response is given a score from 0 to 3 with a maximum total of 30 reflecting the highest degree of impairment.^29^ The Hospital Anxiety and Depression Scale (HADS) was introduced in May 2018.^34^ HADS is a 14‐question self‐rating scale assessing depression (7 items) and anxiety (7 items). A score of ≥11 in either subdomain indicates a high probability of a mood disorder.^34^ Additional exploratory ad hoc questions were included regarding skin concerns.

### 2.3 Clinical assessment

The treating physician assessed participant symptoms that included the number of cNFs (0, 1–19, 20–99, 100–500, >500), presence of facial cNFs, disease severity (Riccardi Scale),^35^ and visibility (Ablon Scale).^36^ The Riccardi Scale grades NF1 severity based on the impact of NF1 on health: grade 1 (mild features with no compromise of health) to grade 4 (intractable compromise of health managed with difficulty).^35^ The Ablon Scale evaluates the visibility of neurofibromatosis in a fully dressed individual: Grade 1, no visible tumors outside normal clothing to Grade 3, severe manifestations of NF1, including facial tumors, optic gliomas, and bone deformities.^36^ Assessments were made in face‐to‐face appointments. A minority of participants (<10), secondary to COVID‐19 lockdown restrictions, attended a telehealth consultation. These participants provided multiple photographs for clinician assessment.

### 2.4 Procedure

Surveys were completed in hard copy before the patient's first skin consultation in the skin clinic waiting room, or via an online survey (hosted by REDCap survey software) from 2020.^37^

### 2.5 Data analysis

Patient demographics and survey scores were evaluated descriptively. Associations between disease visibility, disease severity, age, gender, itch, number, and presence of facial cNFs on QoL, anxiety, and depression scores, were explored using univariate linear regression. To account for potential confounding factors, multivariate linear regression was performed to evaluate the relationship between mental health and QoL scores and age, sex, visibility, and itch. Statistical assumptions underlying regression analysis including independence of observations, linearity, homoscedasticity, outliers, high leverage points, highly influential points, and multicollinearity were assessed for and met. Some of the analysis residuals were not normally distributed, however, residual distributions were symmetrical and did not pose a problem as per the central limit theorem. While multicollinearity was not present, Riccardi score (severity), presence of facial and total number of cNFs are metrics clinically and statistically positively correlated with visibility (Table S5). A washing‐out effect on visibility was seen and these variables were removed from the multivariate linear regression. Skindex‐29, HADS, and DLQI scores were compared to published control populations using independent sample *t*‐tests.^38^, ^39^, ^40^ Control cohorts were selected that had a comparable mean age and gender balance. Pearson correlation coefficients were used to assess correlations between the instruments. Wilcoxon signed‐rank tests were used to compare the median of comparable questions between the surveys. *p*‐values of <0.05 were considered statistically significant. All analyses were performed using SPSS, version 27.0 (IBM Corp.).

## 3 RESULTS

After excluding two patients because of significant language barriers, 105 patients were invited to participate in the study. A total of 100 patients completed the surveys, a 95% response rate. Because of cognitive difficulties, two patients received help from family members to complete the surveys. The mean (standard deviation) age of participants was 42.9 (SD 13.0) years; range, 18–78 years and 68% were female (Table 1). Skin‐related QoL and severity scores from the first 40 patients were published previously.^7^

**TABLE 1:** Demographic and clinical characteristics of NF1 patient cohort.

Characteristics	Patients, *n* (%)
Gender	
Male	32 (32)
Female	68 (68)
Age categories	
<30	13 (13.0)
30–40	37 (37.0)
41–50	26 (26.0)
51–60	12 (12.0)
>60	12 (12.0)
Mean (years) ± SD	42.85 ± 12.89
Number of CNFs^a^	
<20	18 (18.8)
20–99	29 (30.2)
100–500	18 (18.8)
>500	31 (32.3)
Mean ± SD	20–99 ± 1 category
Facial CNFs^a^	
Yes	61 (61.6)
No	38 (38.4)
NF1 severity^a^	
Grade 1	10 (10.3)
Grade 2	39 (40.2)
Grade 3	38 (39.2)
Grade 4	10 (10.3)
Mean ± SD	2.5 ± 0.8
NF1 visibility	
Grade 1	49 (49.0)
Grade 2	30 (30.0)
Grade 3	21 (21.0)
Mean ± SD	1.7 ± 0.8
Itch symptoms	
Yes	42 (42.0)
No	58 (58.0)

Abbreviations: cNF, cutaneous neurofibromas; NF1, neurofibromatosis; SD, standard deviation.

^a^ Decreased number of respondents due to missing data or non‐response (*n* = 97–99).

### 3.1 Health‐related QoL

Impaired QoL was observed in NF1 patients compared to published control populations in all Skindex‐29 domains and in the DLQI (Table S2). Using the proposed clinically relevant impairment cut‐off scores for Skindex‐29,^41^ the mean emotion subdomain score was in the “severely impaired” QoL category (mean [SD]: 44.42 [29.74]) (Table 2). Compared to a general NF1 Australian population, our cohort had more QoL impairment as measured by Skindex‐29 (Table 2). Clinically relevant NF1‐AdQOL control values are not available. Our cohort had higher levels of anxiety and HADS Total scores compared to a control population (Table S2), with 23% exhibiting likely clinical anxiety.

**TABLE 2:** Skindex‐29 and HADS scores by clinical impairment category.

Skindex‐29 domains	Skindex‐29 quality of life impairment category^a^ compared to a general NF1 population			
	No. (%), [% of NF1 population]^b^			
	Less than mild	Mild	Moderate	Severe
Symptoms	62 (62), [88]	6 (6), [2]	10 (10), [3]	22 (22), [7]
Emotions	32 (32), [63]	9 (9), [7]	6 (6), [4]	53 (53), [27]
Functions	57 (57), [76]	10 (10), [6]	2 (2), [2]	31 (31), [15]

	HADS Anxiety and Depression by clinical cut‐off scores^c^		
	*n* (%)		
	Normal (cut off score 0–7)	Borderline (cut off score 8–10)	Abnormal (cut off score > 11)
Anxiety	32 (53.3)	14 (23.3)	14 (23.4)
Depression	48 (80.0)	9 (14.9)	3 (5.1)

Abbreviations: HADS, Hospital Anxiety and Depression Scale; NF1, neurofibromatosis 1.

^a^ Impairment cut‐off scores as per Prinsen et al. 2011.

^b^ Results in percent from Crawford et al. 2022.

^c^ HADS cut off scores as per Zigmond and Snaith 1983.

#### 3.1.1 Factors associated with QoL and well‐being

In Skindex‐29 and NF1‐AdQOL univariate models, the number of cNFs, visibility, gender, severity, and itch were each seen to be significantly associated with one or more survey subdomains (Tables 3 and S3a). In DLQI univariate models, the number of cNFs, visibility, and severity significantly predicted worsening DLQI scores (Table S3b). Itch and gender were not associated with worsening QoL as measured by DLQI scores. Itch and being female were associated with higher anxiety and HADS Total scores (Table S3b). Age was not associated with any QoL, DLQI, anxiety, or depression scores.

**TABLE 3:** Univariate regression analysis demonstrating unadjusted association between demographic/clinical variables and Skindex‐2 scores.^a^

Variable	Univariate analysis Skindex‐29 symptoms			Univariate analysis Skindex‐29 emotions			Univariate analysis Skindex‐29 functioning			Univariate analysis Skindex‐29 total		
	Mean (SD) Skindex symptoms	Coefficient (95% CI)	*p*‐value	Mean (SD) Skindex emotions	Coefficient (95% CI)	*p*‐value	Mean (SD) Skindex functioning	Coefficient (95% CI)	*p*‐value	Mean Skindex‐29 total	Coefficient (95% CI)	*p*‐value
Age (years)^b^												
20–29	27.75 (25.89)	2.812 (−1.23 to 6.85)	0.170	34.42 (28.94)	−1.314 (−6.29 to 3.66)	0.602	13.62 (17.44)	1.000 (−3.35 to 5.35)	0.649	75.79 (64.71)	2.210 (−9.85–14.26)	0.717
30–39	30.12 (23.88)			52.30 (27.65)			30.87 (26.35)			113.28 (66.61)		
40–49	34.62 (20.72)			44.23 (28.76)			26.01 (27.07			104.86 (71.23)		
50–59	37.50 (25.00)			31.12 (26.99)			18.06 (23.29)			86.68 (69.14)		
60+	37.50 (32.03)			44.43 (37.67)			28.75 (32.64)			110.88 (98.25)		
Gender												
Male	22.99 (19.67)	Ref	**0.005**	35.53 (29.56)	Ref	0.040	18.52 (22.21)	Ref	0.076	77.03 (61.55)	Ref	**0.014**
Female	37.34 (25.10)	14.352 (4.35–24.36)		48.60 (29.11)	13.073 (−0.63–25.52)		28.89 (27.51)	9.958 (−1.07 to 20.98)		114.83 (73.77)	37.80	
Facial cNFs												
Absent	26.88 (21.82)	Ref	0.051	40.99 (28.64)	Ref	0.317	18.80 (22.59)	Ref	**0.027**	86.67 (62.05)	Ref	0.065
Present	36.71 (25.31)	9.831 (−0.03 to 19.6)		47.16 (30.26)	6.170 (−5.99 to 18.33)		30.21 (27.61)	11.88 (1.41–22.36)		114.07 (75.99)	27.40 (−1.72 to 56.52)	
No. of cNFs^b^												
<20	26.79 (21.33)	5.934 (1.57–10.29)	**0.008**	27.50 (24.07)	8.273 (3.04–13.51)	**0.002**	11.45 (17.67)	8.314 (3.85–12.78)	**0.000**	65.74 (47.38)	22.35 (9.82–34.88)	**0.001**
20–99	26.73 (23.82)			43.53 (30.47)			23.77 (26.50)			94.04 (75.07)		
100–500	30.16 (27.40)			45.89 (26.85)			24.30 (25.10)			100.34 (66.96)		
>500	43.20 (23.82)			55.50 (31.39)			38.28 (27.37)			136.98 (76.10)		
Visibility^b^												
1	27.04 (21.65)	9.064 (3.18–14.94)	**0.003**	38.16 (28.14)	7.271 (−0.11–14.65)	0.053	19.51 (23.81)	8.693 (2.28–15.10)	**0.008**	84.72 (63.73)	25.15 (7.65–42.66)	**0.005**
2	32.62 (22.68)			49.77 (29.06)			27.09 (24.53)			109.48 (66.78)		
3	46.26 (28.13)			51.35 (32.63)			37.54 (30.67)			135.15 (86.48)		
Severity^b^												
1	19.64 (23.53)	10.275 (3.07–14.67)	**0.003**	31.75 (31.47)	4.595 (−2.78 to 11.98)	0.219	15.83 (22.78)	7.135 (0.17–13.56)	**0.030**	67.23 (69.36)	20.43 (2.83–38.03)	**0.023**
2	30.03 (20.87)			44.55 (27.49)			22.86 (23.91)			97.45 (62.83)		
3	32.33 (24.65)			44.51 (31.04)			27.44 (27.78)			104.29 (75.52)		
4	54.64 (26.46)			51.50 (33.90)			39.78 (31.69)			145.92 (88.20)		
Itch												
No	25.86 (23.66)	Ref	**0.000**	40.15 (31.61)	10.164 (−1.68 to 22.01)	0.092	21.69 (23.83)	Ref	0.070	87.70 (70.29)	Ref	**0.013**
Yes	42.26 (22.20)	16.839 (7.59–26.09)		50.31 (26.18)			30.94 (28.75)	9.617 (−0.78 to 29.92)		123.51 (69.89)	35.81 (7.62–64.01)	

Abbreviations: CI, confidence interval; cNFs, cutaneous neurofibromas; DLQI, Dermatology Life Quality Index; HADS, Hospital Anxiety and Depression Scale; NF1‐AdQOL, Neurofibromatosis 1‐adult quality‐of‐life questionnaire; SD, standard deviation.

^a^ The univariate regression analysis for all surveys did not fit in one table. See Tables S3a and S4b for univariate regression analysis between demographic/clinical variables and NF1‐AdQOL, DLQI, and HADS.

^b^ Variable fitted as continuous variable in regression modeling. Unit of measurement is per 10 year increase for age, and per 1 unit increase for number of cNFs, visibility, and severity score.

In the univariate regression models, itch was the most important clinical factor correlating with NF1‐AdQOL and HADS Total scores. The number of cNFs was the clinical factor correlating most closely with skin‐related QoL (Skindex‐29). Visibility was the key clinical factor correlating with DLQI scores (Table 4).

**TABLE 4:** Proportion of variance in the survey scores explained by the clinical factor (*R* ^2^).^a^

	NF1‐AdQOL	Skindex‐29	DLQI	HADS (total score)
Age	0.012	0.001	0.003	0.003
Gender	0.069	0.061	0.007	0.070
#cNFs	0.071	**0.118**	0.080	0.002
Facial cNFs	0.012	0.035	0.007	0.046
Severity	0.037	0.053	0.073	0.010
Visibility	0.027	0.077	**0.096**	0.011
Itch	**0.087**	0.061	0.013	**0.087**

Abbreviations: cNFs, cutaneous neurofibromas; DLQI, Dermatology Life Quality Index; HADS, Hospital Anxiety and Depression Scale; NF1‐AdQOL, Neurofibromatosis 1‐adult quality‐of‐life questionnaire.

^a^ *R* ^2^ is a measure of the *proportion of variance* in the dependent variable that is explained by the independent variable.

After controlling for age, gender, visibility and itch, increasing visibility significantly predicted worse QoL scores in Skindex‐29 (all subdomains and Total), NF1‐AdQOL (physical symptoms and functioning subdomains and Total), and the DLQI (*p* < 0.05, Table 5). Itch significantly predicted worse QoL in Skindex‐29 (physical symptoms subdomain and Total score) and NF1‐AdQOL (physical symptoms and emotions subdomains and Total score). Being female significantly predicted worse QoL as measured by Skindex‐29 physical symptoms, Skindex‐29 Total, NF1‐AdQOL symptoms, NF1‐AdQOL emotions, NF1‐AdQOL Total, and HADS Anxiety. Age was not associated with increasing QoL scores.

**TABLE 5:** Multivariate regression analysis.

Variable	Skindex‐29 symptoms		Skindex‐29 emotions		Skindex‐29 functions		Skindex‐29 total		NF1‐AdQOL symptoms		NF1‐AdQOL emotions		NF1‐AdQOL functions		NF1‐AdQOL total		HADS anxiety		HADS depression		HADS total		DLQI	
	Coefficient (95% CI)	*p*‐value	Coefficient (95% CI)	*p*‐value	Coefficient (95% CI)	*p*‐value	Coefficient (95% CI)	*p*‐value	*p*‐value	*p*‐value	Coefficient (95% CI)	*p*‐value	Coefficient (95% CI)	*p*‐value	Coefficient (95% CI)	*p*‐value	Coefficient (95% CI)	*p*‐value	Coefficient (95% CI)	*p*‐value	Coefficient (95% CI)	*p*‐value	Coefficient (95% CI)	*p*‐value
Age (years)^a^																								
20–29	1.328 (−2.38–5.03)	0.065	−2.728 (−7.67–2.215)	0.276	−0.855 (−5.17 to 3.46)	0.695	−2.256 (−13.71 to 9.20)	0.697	−1.834 (−5.78 to 2.12)	0.359	−2.880 (−7.19 to 1.43)	0.188	−2.724 (−7.09 to 1.64)	0.218	−9.552 (−20.77 to 1.67)	0.094	−0.160 (−1.06 to 0.74)	0.722	0.391 (−0.40 to 1.18)	0.326	0.232 (−1.28 to 1.74)	0.759	−0.833 (−2.00 to 0.332)	0.158
30–39																								
40–49																								
50–59																								
60+																								
Gender																								
Male	Ref		Ref		Ref		Ref		Ref		Ref		Ref		Ref		Ref		Ref		Ref		Ref	
Female	11.722 (2.325–21.20)	**0.015**	11.653 (−0.89 to 24.20)	0.068	9.256 (1.71–20.22)	0.097	32.63 (3.55–61.71)	**0.028**	11.891 (2.00–21.78)	**0.019**	13.461 (2.66–24.26)	**0.015**	9.523 (−1.40 to 20.45)	0.087	31.57 (3.25–59.90)	**0.029**	2.469 (0.21–4.90)	**0.048**	0.225 (−1.93 to 2.38)	0.835	2.69 (−1.42–6.79)	0.195	0.397	0.789
Visibility^a^																								
1	9.195 (3.60–14.79)	**0.002**	8.872 (1.40–16.34)	**0.020**	9.599 (3.07–16.13)	**0.004**	27.67 (10.35–44.98)	**0.002**	7.832 (1.94–13.72)	**0.010**	6.161 (−0.27 to 12.59)	0.060	7.868 (1.36–14.38)	**0.018**	19.55 (2.71–36.39)	**0.023**	0.004 (−1.49 to 1.50)	0.996	0.686 (−0.64 to 2.01)	0.304	0.690 (−1.83 to 3.21)	0.585	3.220 (1.32–5.12)	**0.001**
2																								
3																								
Itch																								
No	Ref		Ref		Ref		Ref		Ref		Ref		Ref		Ref		Ref		Ref		Ref		Ref	
Yes	14.232 (5.359–23.11)	**0.002**	8.052 (−2.83 to 21.01)	0.180	7.683 (−2.67 to 18.03)	0.144	29.97 (2.51–57.42)	**0.033**	11.605 (2.286–20.92)	**0.015**	11.063 (0.89–21.24)	**0.033**	9.571 (−0.73 to 19.87)	0.068	35.52 (8.83–62.20)	**0.010**	1.457 (−0.88 to 3.80)	0.217	1.606 (−0.46–3.67)	0.125	3.063 (−0.87 to 7.00)	0.125	1.463 (−1.25 to 4.18)	0.287

Abbreviations: CI, confidence interval; HADS, Hospital Anxiety and Depression Scale; DLQI, Dermatology Life Quality Index; NF1‐AdQOL, Neurofibromatosis 1‐adult quality‐of‐life questionnaire.

^a^ Variable fitted as continuous variable in regression modeling. Unit of measurement is per 10 year increase for age, and per 1 unit increase for number of cutaneous neurofibromas, visibility and severity score.

### 3.2 Survey and variable comparisons

Strong correlations between survey instruments were seen with Skindex‐29 most closely correlated with NF1‐AdQOL (*r* = 0.852, *p* < 0.0001), and to a lesser extent with DLQI (*r* = 0.651, *p* < 0.0001). NF1‐AdQOL also correlated with DLQI scores (*r* = 0.651, *p* < 0.0001) (Table S4).

The highest mean scoring items in both Skindex‐29 and NF1‐AdQOL concerned worry about worsening skin disease and embarrassment. The equal highest mean scoring items in DLQI were “How itchy, sore, painful or stinging has your skin been” and “How embarrassed or self‐conscious have you been because of your skin”.

Mean scores in all subdomains were higher in NF1‐AdQOL compared with Skindex‐29 (Table S5). When comparing individual items with comparable phrasing, there was a significant median increase in item score for NF1‐AdQOL compared to Skindex‐29 and DLQI items (Table S6).

In a univariate regression model, when looking at the two highest scoring survey questions in each survey, “I am embarrassed by the way NF1 affects my physical appearance” (NF1‐AdQOL) was the most important question correlating to HADS anxiety and HADS depression (Table S7). Table S7 displays the association between the highest scoring survey items and mental health scores.

NF1‐AdQOL is the only instrument measuring worry about passing NF1 to children; 58% of our cohort stated they “often” or “all the time” worry about passing on the condition.

## 4 DISCUSSION

Our patient cohort had significantly poorer QoL and mood compared to control populations, consistent with findings in the general NF1 population.^2^, ^42^ NF1‐specific and skin‐related QoL scores were also higher than other NF1 patient cohort studies,^4^, ^5^, ^9^, ^30^ which is not unexpected given these patients were attending a cNF treatment clinic. This cohort is likely to be most representative of those interested in future clinical trials of new NF1 skin treatments and uptake of new therapies.

When exploring how much variance in the survey scores can be explained by the clinical factors collected, cNF burden was the most important clinical factor for Skindex‐29 (Table 4), similar to our previous study.^7^ Reflecting similarly collected metrics, clinician‐rated disease visibility was the most important clinical factor in DLQI. As described previously,^4^, ^5^, ^9^ QoL scores in the emotions domain were consistently higher than in the physical symptoms and functioning domains, reflecting the greater emotional burden of cutaneous features.

Visibility was not associated with anxiety or depression, which is consistent with the findings of Hamoy‐Jimenez^2^ and Wang^13^ that visibility was not indicative of emotional functioning and mental health. This finding juxtaposes several other studies.^6^, ^17^, ^18^ However, this may be due to use of clinician‐rated visibility scoring by Hamoy‐Jimenez, Wang, and this study. Hamoy‐Jimenez et al. found that although examiner‐assessed visibility was not correlated with mental health, self‐reported physical appearance was a major driver of mental‐health‐related QoL.^2^ In this study, mental health scores worsened as the degree of embarrassment with physical disability increased (Table S7), illustrating the powerful impact of an individual's internal perception of disfigurement. Questions relating to embarrassment and worry about disease progression were the highest mean scoring questions in both Skindex‐29 and NF1‐AdQOL, suggesting that it will be essential to include these factors in future PROMs for cNF treatment. Mirroring Wolkenstein's et al.'s paper,^9^ clinician‐assessed disease severity in this study was not indicative of emotional QoL, anxiety, or mood.

There were significantly higher levels of anxiety in our cohort compared to general population levels.^40^ Interestingly, levels of depression in our cohort were the same as general population levels,^40^ which was lower than expected given previous prevalence estimates of 19%–55% of studied NF1 cohorts.^6^, ^13^, ^19^ This may indicate that patients presenting to a skin clinic have more self‐efficacy and agency than general NF1 cohorts.^6^ Anticipation of a reduction in disease disfigurement might also contribute. Notably, however, comparison between depression levels in published NF1 cohorts is challenging given the disparate surveys used (e.g., the EQ‐5D‐5L,^13^ Center for Epidemiologic Studies Depression Scale,^13^, ^19^ and the Patient Health Questionnaire Depression Scale).^6^

Itch was an important predictor of worsening Skindex‐29 and NF1‐related QoL scores. Interestingly, itch was not associated with DLQI (a largely functional based survey) or with the functional domains of QoL instruments, suggesting that itch may not impair functioning. This contrasts with the impact of chronic itch on DLQI scores in dermatology out‐patients.^43^ While the effect size was modest, itch was the most important clinical factor correlating with anxiety, depression, and NF1‐related QoL scores, explaining 8.7% of the variance in scores. Therefore, this study joins a new body of literature detailing the significant impact of itch on QoL in NF1 adults.^16^

Females experienced poorer skin‐specific and NF1‐specific QoL, consistent with previous skin‐specific QoL findings.^4^, ^5^, ^7^ Being female also predicted higher anxiety scores; but not depression scores, perhaps mirroring the higher incidence of anxiety in females compared to males (21% vs 12.4%) in Australia generally.^44^ In previous studies,^4^, ^5^, ^9^ gender was not associated with general HR QoL.

As scores in NF1‐related QoL were higher than skin‐related QoL, we compared items with comparable phrasing and intent and found that NF1‐specific items scored significantly higher than skin‐specific items. Therefore, NF1‐specific questions may be clearer, more relatable, and possibly more sensitive, as also suggested by Hamoy‐Jimenez et al. regarding items related to appearance.^2^ The recently developed cNF Skindex may be suitable for this purpose.^33^

Measures of cNF disease burden are paramount to the design of cNF treatment measures. In this study, the burden of cNFs was determined in three different ways: clinician‐rated visibility using the Ablon scale, clinician‐estimated number of cNFs, and presence or absence of facial cNFs. The Ablon visibility score and clinician‐estimated number of cNFs correlated strongly (Table S1), and we found them to have comparable impacts on QoL scores. Interestingly, the presence of facial neurofibromas did not predict worsening QoL scores in any measure. After controlling for confounders (age, gender and itch), the Ablon score was an important predictor of reduced skin‐related and overall QoL, similar to previous studies.^2^, ^3^, ^4^, ^5^, ^6^, ^7^, ^9^, ^42^ Due to the ease of utility of the Ablon score and current lack of cNF counting automation, we believe collection of both clinician‐ and patient‐rated visibility using the Ablon scale^36^ is an essential and powerful tool for use in future studies.

While not cNF‐specific, the brevity of DLQI and the fact it has been extensively used and was sensitive to visibility changes while not impacted by gender, age or itch, makes it a measure with key strengths relevant to cNF trials.

Despite a national referral base, this research was undertaken in a single, tertiary hospital with a predominance of female patients. Patient‐rated disease visibility was not collected and may better reflect the patient experience and more accurately capture impact of skin disease and treatment on patient's QoL and mental health. In addition, given the large number of items, survey fatigue may have occurred. Further longitudinal studies are required to test for sensitivity to change for each instrument.^32^ Ideally, the use of an NF1‐specific skin disease short‐form PROM, together with objective methods of measuring cNF number and size, to evaluate treatment outcomes, is required.^33^

## 5 CONCLUSION

Disease visibility and itch are associated with poorer skin‐ and NF1‐related QoL in individuals seeking cNF treatment and are, therefore, important to address clinically. We show the large emotional impact of cNF appearance, which highlights the value of patient reported QoL measures evaluating the impact of the emotional burden of the disease and the need for both patient and clinician ratings of visibility in the evaluation of skin treatments. We found higher levels of likely clinical anxiety than previously reported,^6^ suggesting a need for screening, surveillance, and treatment in the design of treatment‐based cosmetic clinical trials and future research studies. NF1‐specific questions may capture the NF1 experience more accurately and skin‐specific QoL items are more likely to capture changes in disease burden relevant to the skin treatments delivered. Outcome measures used in cNF therapeutic trials and further research should include evaluation of visibility, patient‐reported QoL, itch, and anxiety. This work creates a foundation for further research into the sensitivity of individual survey items to detect meaningful treatment outcomes for individuals with bothersome cNFs.

## CONFLICT OF INTEREST STATEMENT

None declared.

## Supporting information


**Data S1.**.

## Data Availability

Data used for analysis can be provided after ethics approval on request.
