# Supplementary material for: The impact of cutaneous neurofibromas on quality of life and mental health in neurofibromatosis type 1
Source: J Dermatol. 2024 Jun 24;51(8):1050–9. doi: 10.1111/1346-8138.17276 (PMC11483957; doi:10.1111/1346-8138.17276)
Supplement: Supplementary file 1 — Data S1.. [file JDE-51--s001.docx]

**Supplementary Material**

SUPPLEMENTARY TABLE 1: Spearman’s rho correlation coefficient between variables (2-tailed)

| Variables | Age | Gender | Facial Neurofibromas | No. of cutaneous neurofibromas | Visibility | Severity | Itch |
| --- | --- | --- | --- | --- | --- | --- | --- |
| Age |  |  |  |  |  |  |  |
| Gender | -0.019 |  |  |  |  |  |  |
| Facial Neurofibromas | 0.336*** | 0.049 |  |  |  |  |  |
| No. of cutaneous neurofibromas | 0.311** | -0.040 | 0.428** |  |  |  |  |
| Visibility | 0.269** | -0.048 | 0.489** | 0.660*** |  |  |  |
| Severity | 0.054 | -0.170 | 0.087 | 0.407*** | 0.511*** |  |  |
| Itch | 0.026 | 0.236* | 0.173 | -0.036 | -0.029 | -0.040 |  |

Significance: *p=<0.05, **p=<0.01, ***p=<0.001

SUPPLEMENTARY TABLE 2. Skin Clinic NF1 score compared to control populations

| Skindex-29 | Skin Clinic NF1 mean ± SD (n = 101) | Danish population§  mean ± SD (n = 255) | t-statistic, p value |
| --- | --- | --- | --- |
| Symptoms | 32.8 ± 24.3 | 5.9 ± 10.6 | 10.5, **<0.0001** |
| Emotions | 44.4 ± 29.7 | 1.9 ± 7.3 | 14.0, **<0.0001** |
| Functions | 25.6 ± 26.3 | 0.9 ± 4.5 | 9.3, **<0.0001** |
| HADS | **Skin Clinic NF1 mean ± SD**  (n = 61) | **Colombian population† mean ± SD**  (n = 1500) | **t-statistic, p value** |
| Anxiety | 7.6 ± 4.2 | 4.6 ± 3.6 | 5.1**, <0.0001** |
| Depression | 4.4 ± 3.6 | 4.3 ± 3.9 | 0.1, <0.932 |
| Total | 12.0 ± 7.0 | 8.9 ± 3.8 | 3.2, **0.001** |
| DLQI | **Skin Clinic NF1 mean ± SD** (n = 77) | **Danish population‡** mean ± SD (n = 100) | **t-statistic, p value** |
| Total | 6.1 ± 6.0 | 0.4 ± 1.3 | 8.2, **<0.0001** |
| NF1-AdQOL | **Skin Clinic NF1-AdQOL** | No control | N/A |
| Symptoms | 47.72±24.18 | No control | N/A |
| Emotions | 58.30±25.97 | No control | N/A |
| Functions | 36.25±25.80 | No control |  |

**† Hinz et al. 2014**

**‡ Zachariae et al. 2000**

**§ Vinding et al. 2014**

SUPPLEMENTARY TABLE 3a: Results of univariate linear regression analysis demonstrating unadjusted association between demographic/clinical variables and NF1-AdQOL domain scores

|  |  | **Univariate analysis NF1-AdQOL Symptoms** | |  | **Univariate analysis NF1-AdQOL Emotions** | |  | **Univariate analysis**  **NF1-AdQOL Functions** | | **Univariate analysis**  **NF1-AdQOL Total** | | |
| --- | --- | --- | --- | --- | --- | --- | --- | --- | --- | --- | --- | --- |
| **Variable:** | Mean NF1-AdQOL Symptoms (SD) | Coefficient (95% CI) | P-value | Mean NF1-AdQOL Emotions (SD) | Coefficient  (95% CI) | P-value | Mean NF1-AdQOL Functions (SD) | Coefficient  (95% CI) | P-value | Mean NF1-AdQOL Total (SD) | Coefficient (95% CI) | P-value |
| **Age (yrs)*:**  **20-29**  **30-39**  **40-49**  **50-59**  **60+** | 41.83 (19.24)  50.63 (22.49)  48.92 (26.26)  48.13 (29.36)  41.76 (29.36) | -0.610  (-4.79-3.57) | 0.773 | 52.25 (24.37)  64.76 (22.40)  57.16 (26.91)  50.21 (30.70)  53.82 (31.66) | -1.96  (-6.43-2.51) | 0.387 | 29.72 (19.18)  43.01 (22.18)  33.12 (29.01)  31.14 (30.89)  33.29 (30.53) | -1.478  (-5.93-2.98) | 0.512 | 123.79 (56.08)  158.40 (55.65)  139.20 (75.81)  129.47 (83.10)  118.13 (90.45 | -6.25  (-17.96-5.46) | 0.292 |
| **Gender:**  **Male**  **Female** | 38.10 (25.00)  52.25 (22.58) | Ref  14.14  (4.04-24.24) | **0.007** | 47.54 (27.17)  63.36 (23.95) | 15.83  (5.02-26.64) | **0.005** | 28.54 (24.09)  39.88 (25.96) | 11.359  (-0.39-22.33) | **0.043** | 114.16 (69.56)  153.17 (66.33) | Ref  39.01  (9.97-68.05) | **0.009** |
| **Facial cNFs:**  **Absent**  **Present** | 46.25 (22.60)  49.19 (25.19) | Ref  2.94  (-7.09-12.97) | 0.562 | 55.23 (24.13)  60.90 (26.92) | Ref  5.667  (-5.05-16.38) | 0.296 | 30.93 (20.52)  40.33 (28.14) | Ref  9.400  (-1.13-19.93) | 0.080 | 132.41 (57.48)  147.86 (75.53) | Ref  15.46  (-13.06-43.98) | 0.285 |
| **No. of cNFs*:**  **<20**  **20-99**  **100-500**  **>500** | 33.46 (20.43)  48.42 (21.49)  43.75 (23.90)  55.31 (26.44) | 5.814  (1.45-10.17) | **0.010** | 39.58 (25.79)  59.41 (25.61)  62.65 (18.81)  65.56 (27.48) | 7.149  (2.45-11.84) | **0.003** | 24.20 (21.74)  36.65 (27.08)  35.96 (20.98)  44.74 (28.28) | 5.837  (1.125-10.55) | **0.016** | 97.24 (63.20)  144.49 (65.70)  142.36 (54.90)  160.27 (79.64) | 16.88  (4.27-29.49) | **0.009** |
| **Visibility*:**  **1**  **2**  **3** | 42.74 (22.37)  50.32 (20.15)  55.94 (31.18) | 6.738  (0.66-12.82) | **0.030** | 53.25 (25.43)  65.46 (23.96)  60.06 (28.55) | 4.633  (-2.00-11.26) | 0.168 | 31.64 (24.13)  38.04 (22.68)  44.73 (32.13) | 6.530  (-0.11-13.05) | **0.050** | 127.62 (63.31)  153.82 (56.70)  153.08 (92.99) | 14.48  (-2.94-31.89) | **0.102** |
| **Severity*:**  **1**  **2**  **3**  **4** | 26.88 (20.10)  45.51 (21.29)  50.35 (25.75)  61.88 (21.49) | 9.665  (3.94-15.39) | **0.001** | 38.54 (28.52)  63.37 (20.81)  58.01 (28.79)  54.65 (25.69) | 2.199  (-4.32-8.72) | 0.505 | 23.41 (21.59)  36.25 (25.80)  35.96 (25.23)  47.41 (32.65) | 5.583  (-0.868-12.03) | 0.089 | 88.83 (62.25)  145.13 (61.35)  140.41 (74.31)  163.94 (77.47) | 16.26  (-0.91-33.43) | 0.063 |
| **Itch:**  **No**  **Yes** | 41.80 (24.23)  55.82 (21.91) | 14.03  (4.53-23.52) | **0.004** | 52.48 (27.62)  66.27 (21.37) | 13.79  (3.51-24.06) | **0.009** | 31.42 (23.48)  42.86 (27.61) | 11.442  (1.12-21.77) | **0.030** | 123.48 (68.91)  164.95 (63.41) | Ref  41.46  (14.36-68.57) | **0.003** |

*Variable fitted as continuous variable in regression modelling. Unit of measurement is per 10 year increase for age, and per 1 unit increase for number of cNFs, visibility and severity score.

SUPPLEMENTARY TABLE 3b: Results of univariate linear regression analysis demonstrating unadjusted association between demographic/clinical variables and DLQI and HADS scores

|  |  | **HADS Anxiety** | |  | **HADS Depression** | |  | **HADS Total** | |  | **DLQI** | |
| --- | --- | --- | --- | --- | --- | --- | --- | --- | --- | --- | --- | --- |
| **Variable:** | Mean HADS Anxiety (SD) | Coefficient (95% CI) | P-value | Mean HADS Depression (SD) | Coefficient  (95% CI) | P-value | Mean HADS Total (SD) | Coefficient  (95% CI) | P-value | Mean DLQI (SD) | Coefficient (95% CI) | P-value |
| **Age (yrs)*:**  **20-29**  **30-39**  **40-49**  **50-59**  **60+** | 7.00 (4.36)  8.55 (4.05)  6.87 (4.44)  7.44 (3.61)  7.14 (5.18) | -0.185  (-1.08-0.71) | 0.680 | 3.22 (3.93)  4.40 (3.03)  4.20 (3.55)  4.78 (3.60)  5.71 (5.28) | 0.479  (-0.28-1.24) | 0.214 | 10.22 (7.16)  12.95 (6.07)  11.07 (7.50)  12.22 (6.44)  12.86 (9.63) | 0.294  (-1.19-1.78) | 0.693 | 4.00 (3.56)  8.26 (7.00)  5.19 (5.03)  4.33 (3.32)  6.33 (7.87) | -0.251  (-1.41-0.91) | 0.668 |
| **Gender:**  **Male**  **Female** | 5.47 (3.31)  8.54 (4.23) | Ref  3.06  (0.86-5.27) | **0.007** | 3.79 (2.82)  4.66 (3.93) | Ref  0.869  (-1.15-2.88) | 0.391 | 9.26 (5.03)  13.20 (7.40) | Ref  3.932  (0.18-7.69) | **0.040** | 5.39 (4.75)  6.49 (6.47) | Ref  1.099  (-1.89-4.10) | 0.466 |
| **Facial cNFs:**  **Absent**  **Present** | 6.61 (3.71)  8.15 (4.26) | Ref  1.535  (-0.79-3.86) | 0.191 | 3.33 (2.77)  4.95 (3.84) | Ref  1.618  (-0.39-3.63) | 0.113 | 9.94 (5.00)  13.10 (7.38) | Ref  3.153  (-0.67-6.98) | 0.104 | 5.08 (5.199)  6.86 (6.33) | Ref  1.78  (-1.11-4.67) | 0.223 |
| **No. of cNFs*:**  **<20**  **20-99**  **100-500**  **>500** | 6.17 (4.63)  8.84 (4.14)  6.56 (3.20)  7.46 (4.65) | 0.045  (-1.04-1.13) | 0.934 | 3.83 (4.13)  4.63 (3.37)  3.81 (3.75)  5.27 (3.63) | 0.291  (-0.65-1.23) | 0.538 | 10.00 (8.41)  13.47 (6.27)  10.37 (5.96)  12.73 (7.88) | 0.336  (-1.48-2.15) | 0.712 | 3.73 (4.74)  5.95 (6.20)  6.00 (5.64)  8.90 (6.59) | 1.556  (0.31-2.80) | **0.015** |
| **Visibility*:**  **1**  **2**  **3** | 7.46 (4.42)  8.00 (4.13)  7.11 (3.80) | -0.012  (-1.50-1.47) | 0.987 | 3.85 (3.36)  4.56 (2.87)  6.00 (5.43) | 0.994  (-0.26-2.25) | 0.119 | 11.30 (6.82)  12.56 (6.34)  13.11 (8.99) | 0.981  (-1.47-3.43) | 0.426 | 4.93 (5.65)  5.80 (4.93)  11.45 (7.03) | 2.694  (0.90-4.49) | **0.004** |
| **Severity*:**  **1**  **2**  **3**  **4** | 5.44 (2.30)  8.61 (4.57)  7.62 (4.04)  6.50 (6.03) | 0.284  (-1.09-1.66) | 0.680 | 3.33 (3.00)  4.65 (3.58)  4.33 (3.23)  6.25 (7.76) | 0.570  (-0.62-1.76) | 0.341 | 8.78 (4.52)  13.26 (7.16)  11.95 (6.45)  12.75 (13.67) | 0.854  (-1.43-3.14) | 0.457 | 2.37 (2.77)  5.66 (5.83)  6.84 (5.96)  9.88 (7.88) | 2.128  (0.49-3.77) | **0.012** |
| **Itch:**  **No**  **Yes** | 6.13 (3.69)  8.56 (4.27) | Ref  2.40  (0.27-4.54) | **0.028** | 3.33 (3.25)  5.08 (3.72) | Ref  1.750  (-0.12-3.62) | 0.066 | 9.46 (5.91)  13.61 (7.17) | Ref  4.153  (0.62-7.69) | **0.022** | 5.53 (5.76)  6.86 (6.25) | Ref  1.336  (-1.41-4.08) | 0.335 |

*Variable fitted as continuous variable in regression modelling. Unit of measurement is per 10 year increase for age, and per 1 unit increase for number of cNFs, visibility and severity score.

SUPPLEMENTARY TABLE 4. Pearson correlation between instruments (2-tailed)

|  | | | | | |
| --- | --- | --- | --- | --- | --- |
|  | Skindex Total | NF1-AdQOL Total | HADS Total | DQLI Total |  |
| Skindex Total | 1 |  |  |  |  |
| NF1-AdQOL Total | 0.852*** | 1 |  |  |  |
| HADS Total | 0.640*** | 0.730*** | 1 |  |  |
| DLQI Total | 0.799*** | 0.651*** | 0.503*** | 1 |  |
|  | | | | | |

Significance: *p=<0.05, **p=<0.01, ***p=<0.001

SUPPLEMENTARY TABLE 5. Summary of surveys scores

|  | Skindex-29  Symptoms | Skindex-29  Emotions | Skindex-29  Functions | Skindex-29 Total | NF1-AdQOL  Symptoms | NF1-AdQOL  Emotions | NF1-AdQOL  Functions | NF1-AdQOL  Total | HADS Anxiety | HADS Depression | HADS Total | DLQI |
| --- | --- | --- | --- | --- | --- | --- | --- | --- | --- | --- | --- | --- |
| N | 100 | 100 | 100 | 100 | 97 | 97 | 97 | 97 | 60 | 60 | 60 | 76 |
| Mean | 32.75 | 44.42 | 25.57 | 102.74 | 47.72 | 58.30 | 36.25 | 140.83 | 7.57 | 4.38 | 11.95 | 6.16 |
| Median | 28.57 | 40.00 | 17.71 | 87.71 | 50.00 | 61.36 | 31.82 | 140.73 | 7.00 | 4.00 | 11.00 | 4.00 |
| Std. Deviation | 24.35 | 29.74 | 26.27 | 71.996 | 24.18 | 25.97 | 25.80 | 69.44 | 4.19 | 3.62 | 7.01 | 6.00 |

SUPPLEMENTARY TABLE 6. Survey question comparisons

|  | **Comparable survey questions** | **Percent selecting “often” or “all the time” (Skindex-29 and NF1-AdQOL) or “a lot” or “very much” (DLQI)**  Percent (mean) | **Median difference** | **T-test,**  **P-value** |
| --- | --- | --- | --- | --- |
| 1 | “I worry that my skin condition may get worse” – Skindex-29 | 48.5% (62.50) | 25 | -3.27, **0.001** |
|  | “I worry that the NF1 lumps will grow bigger” - NF1-AdQOL | 71.1% (76.80) |  |  |
| 2 | “I am embarrassed by my skin condition” – Skindex-29 | 39% (48.74) | 25 | -2.39, **0.017** |
|  | “I am embarrassed by the way my NF1 affects my physical appearance” - NF1-AdQOL | 51.6% (60.31) |  |  |
|  | “Over the last week, how embarrassed or self-conscious have you been because of your skin” – DLQI† | 27.6% (1.17) | N/A | -7.01, **<0.001** |
| 3 | “My skin hurts” – Skindex-29 | 19.8% (36.01) | 25 | -2.16, **0.030** |
|  | “I have NF1 lumps that hurt” - NF1-AdQOL | 30.9% (45.10) |  |  |
| 4 | “My skin is irritated” – Skindex-29 | 22.8% (38.75) | 11.46 | -3.76, **<0.001** |
|  | “My NF1 lumps are physically irritating” - NF1-AdQOL | 42.2% (55.15) |  |  |

† Question compared to NF1-AdQOL comparative question though note DLQI has a one week-recall

SUPPLEMENTARY TABLE 7: Association between key survey items and Mental Health (HADS Anxiety and HADS Depression)

|  | HADS Anxiety | | | HADS Depression | | |  |  |
| --- | --- | --- | --- | --- | --- | --- | --- | --- |
|  | Coefficient (95% CI) | P-value | R^2^ | Coefficient (95% CI) | P-value | R^2^ |  |  |
| I am embarrassed by the way NF1 affects my physical appearance (NF1-AdQOL)† | 0.056  (0.030-0.082) | **<0.001** | **0.244** | 0.058  (0.036-0.079) | **<0.001** | **0.336** |  |  |
| I worry about getting more NF1 lumps as I get older (NF1-AdQOL) | 0.075  (0.036-0.113) | **<0.001** | 0.218 | 0.066  (0.032-0.099) | **<0.001** | 0.220 |  |  |
| I worry that my skin condition may get worse (Skindex-29) | 0.045  (0.010-0.079) | **0.012** | 0.107 | 0.041  (0.012-0.71) | **0.007** | 0.120 |  |  |
| I am embarrassed by my skin condition (Skindex-29) | 0.041  (0.014-0.069) | **0.004** | 0.135 | 0.053  (0.031-0.075) | **<0.001** | 0.295 |  |  |
| Over the last week, how itchy, sore, painful or stinging has your skin been? (DQLI) | 0.088  (-0.015-2.192) | 0.053 | 0.069 | 1.158  (0.25702.058) | **0.013** | 0.111 |  |  |
| Over the last week, how embarrassed or self-conscious have you been because of your skin? (DQLI) | 1.333  (0.161-2.506) | **0.027** | 0.089 | 1.562  (0.630-2.493) | **<0.001** | 0.176 |  |  |

† Unit of measurement is per 1 unit increase in embarrassment from a 5-scale gradient.
